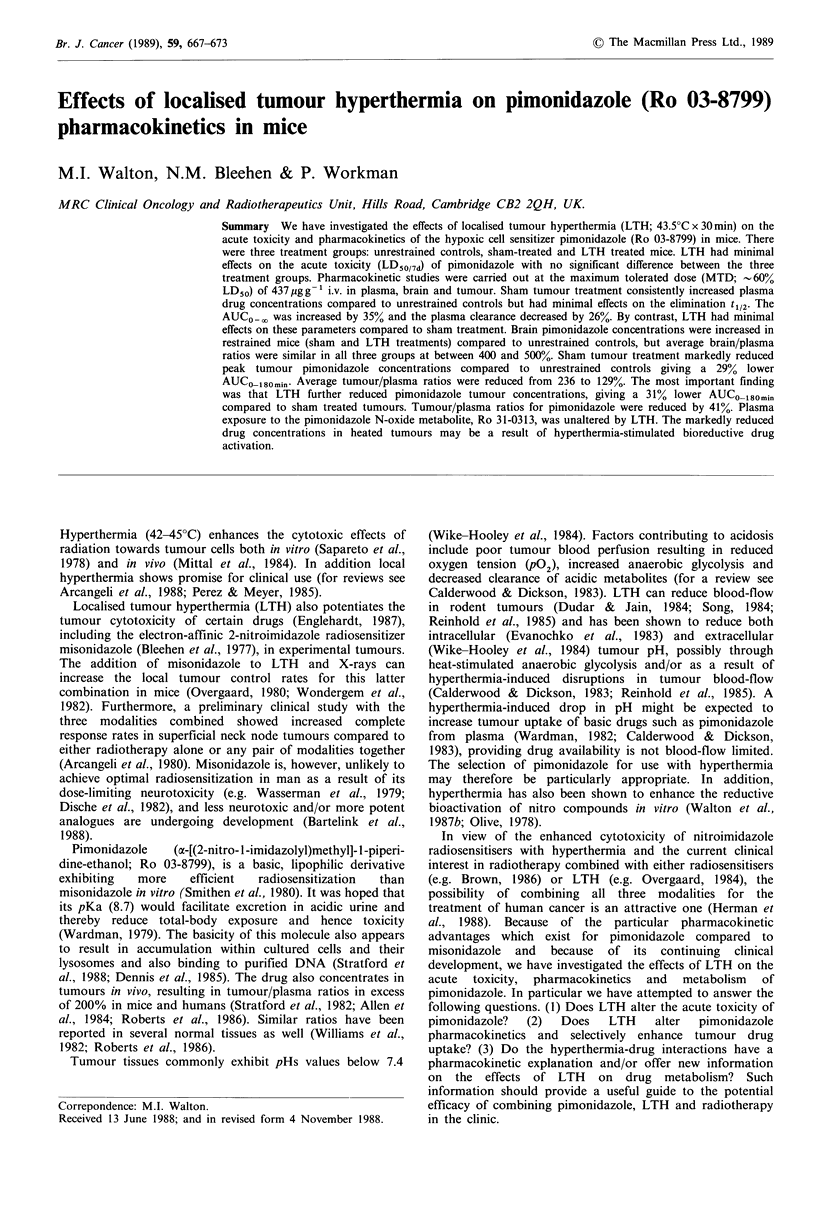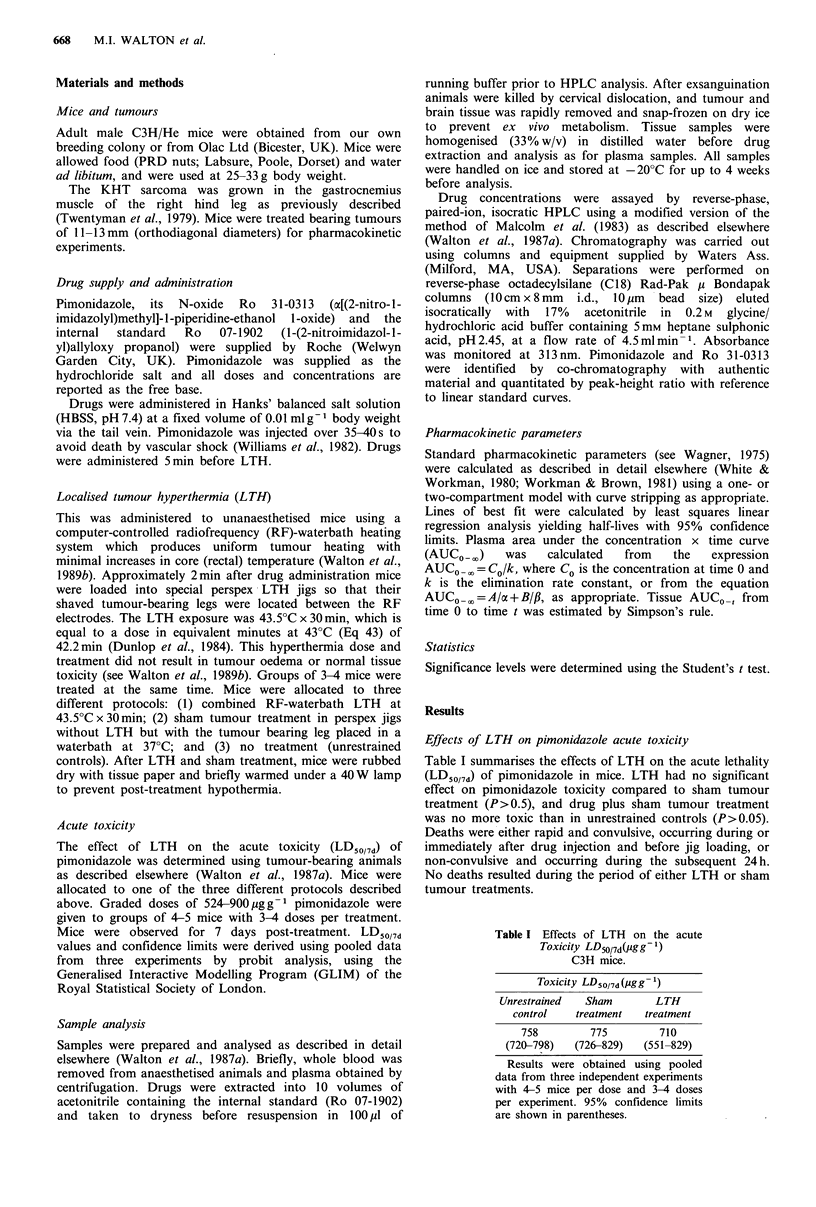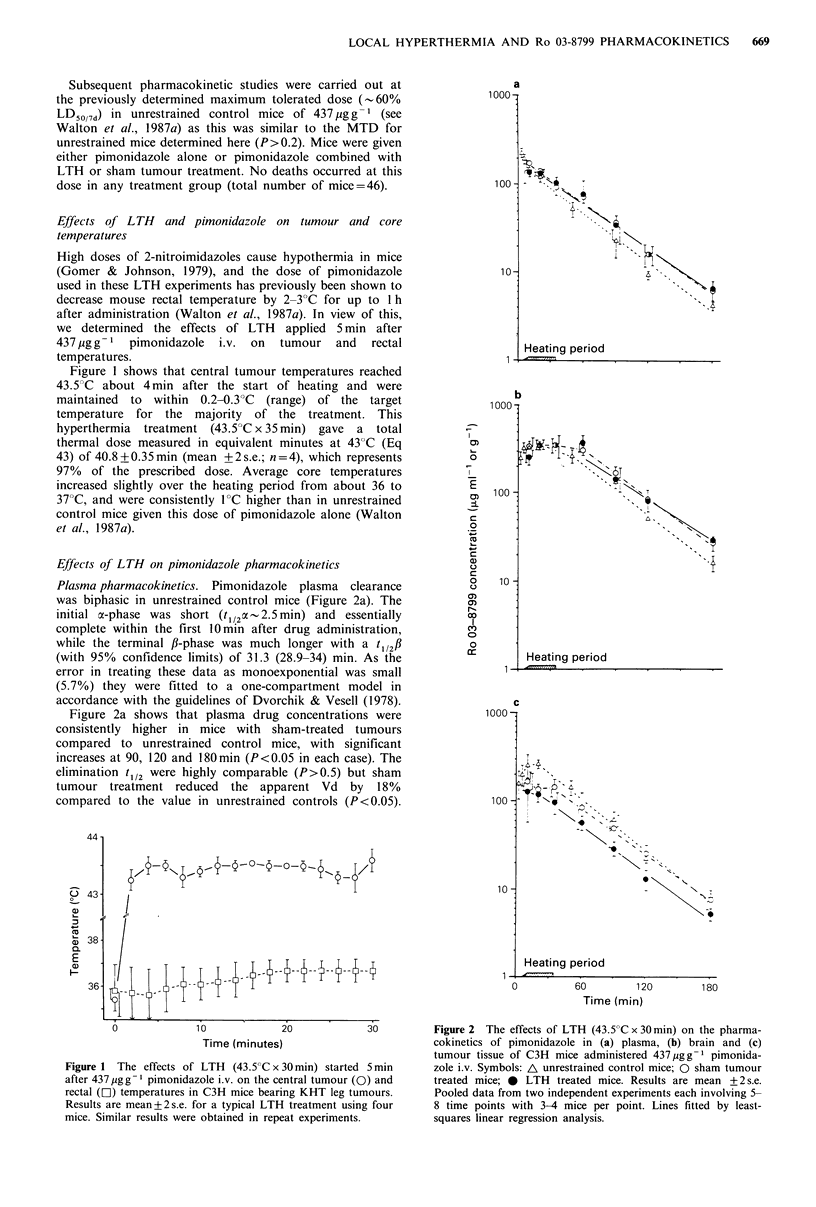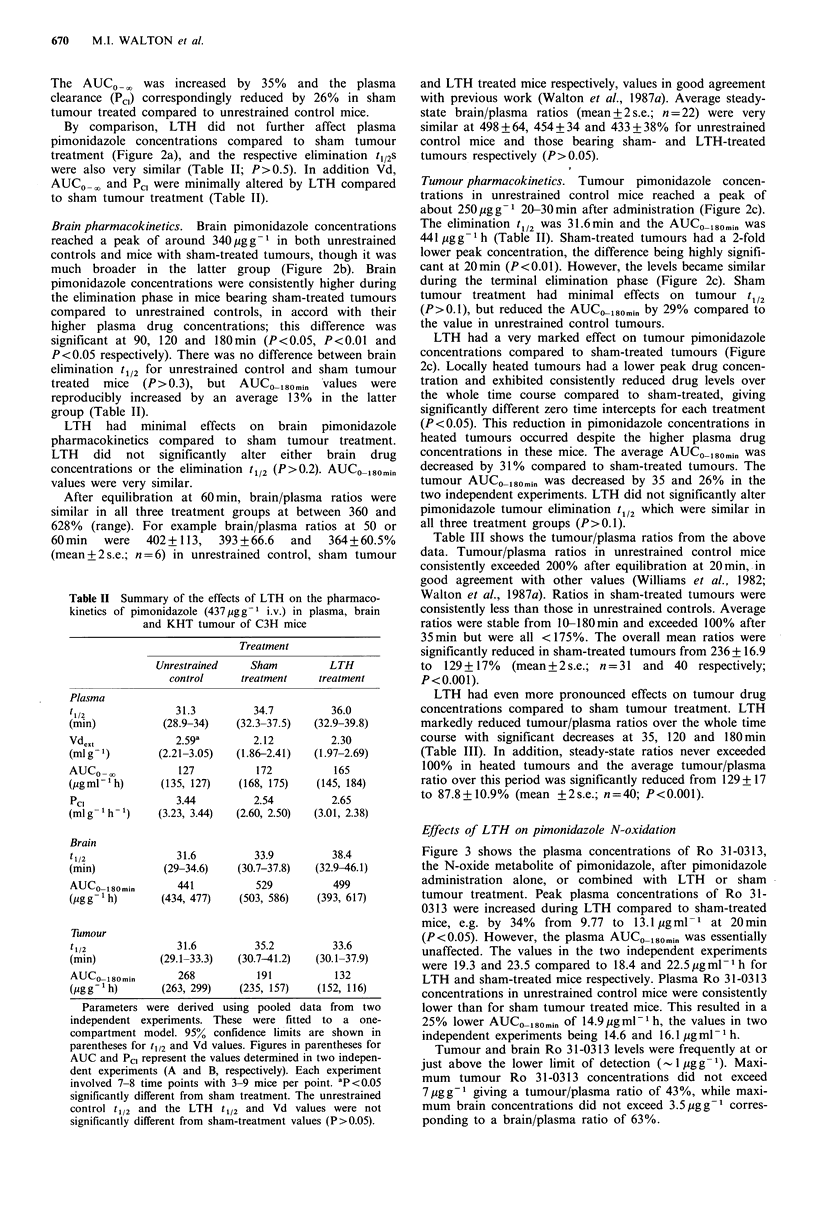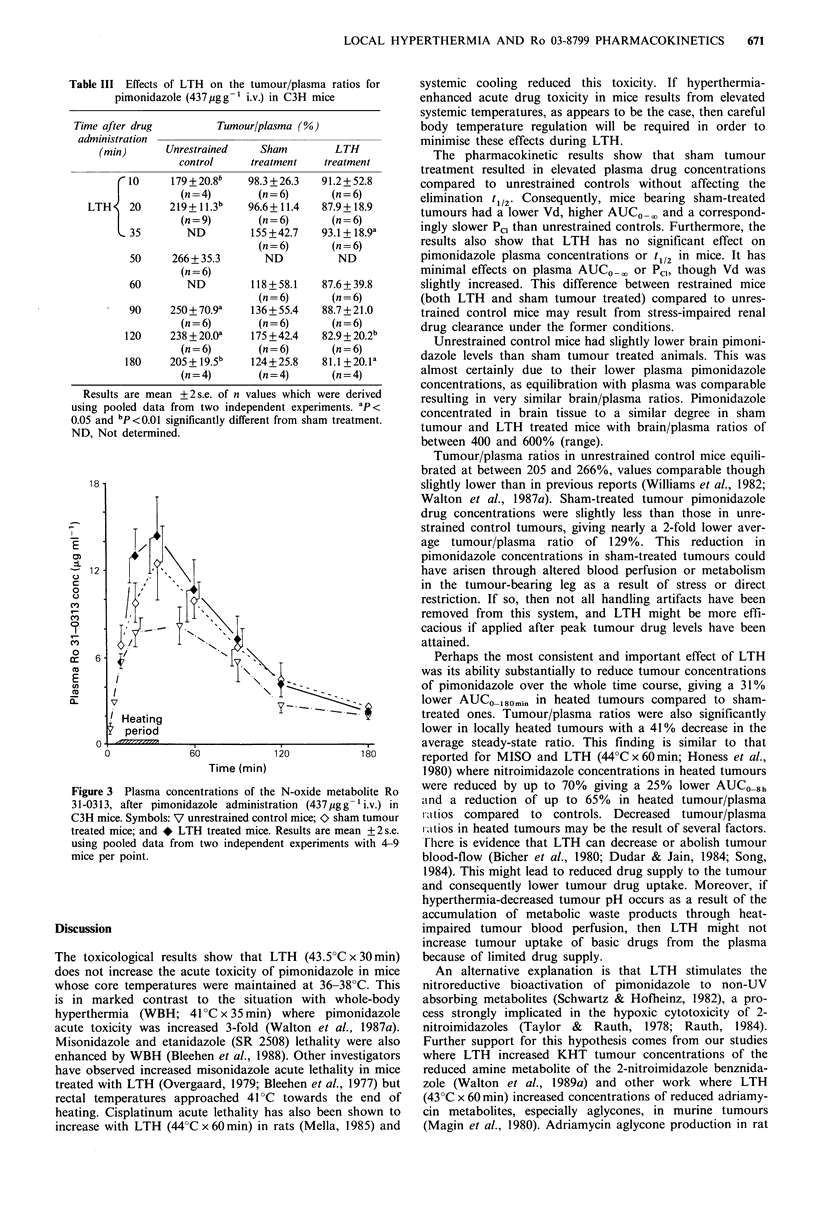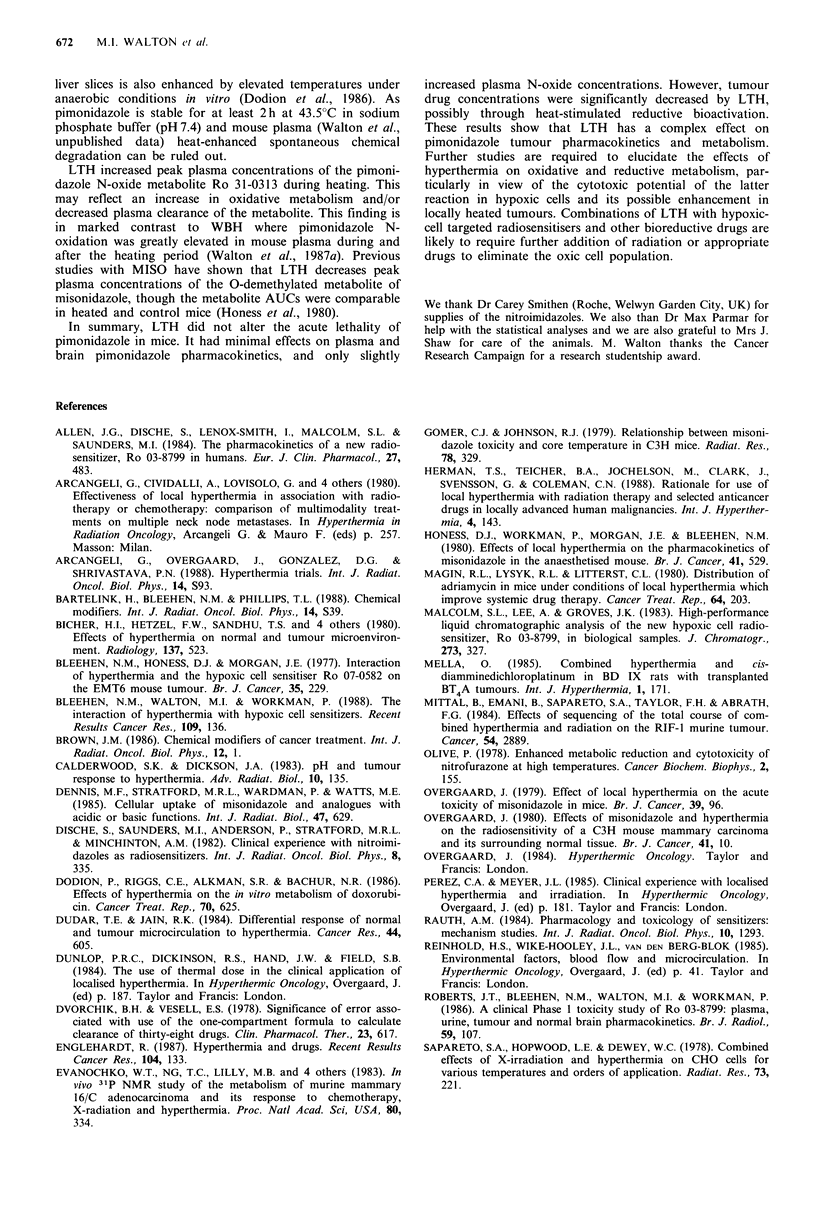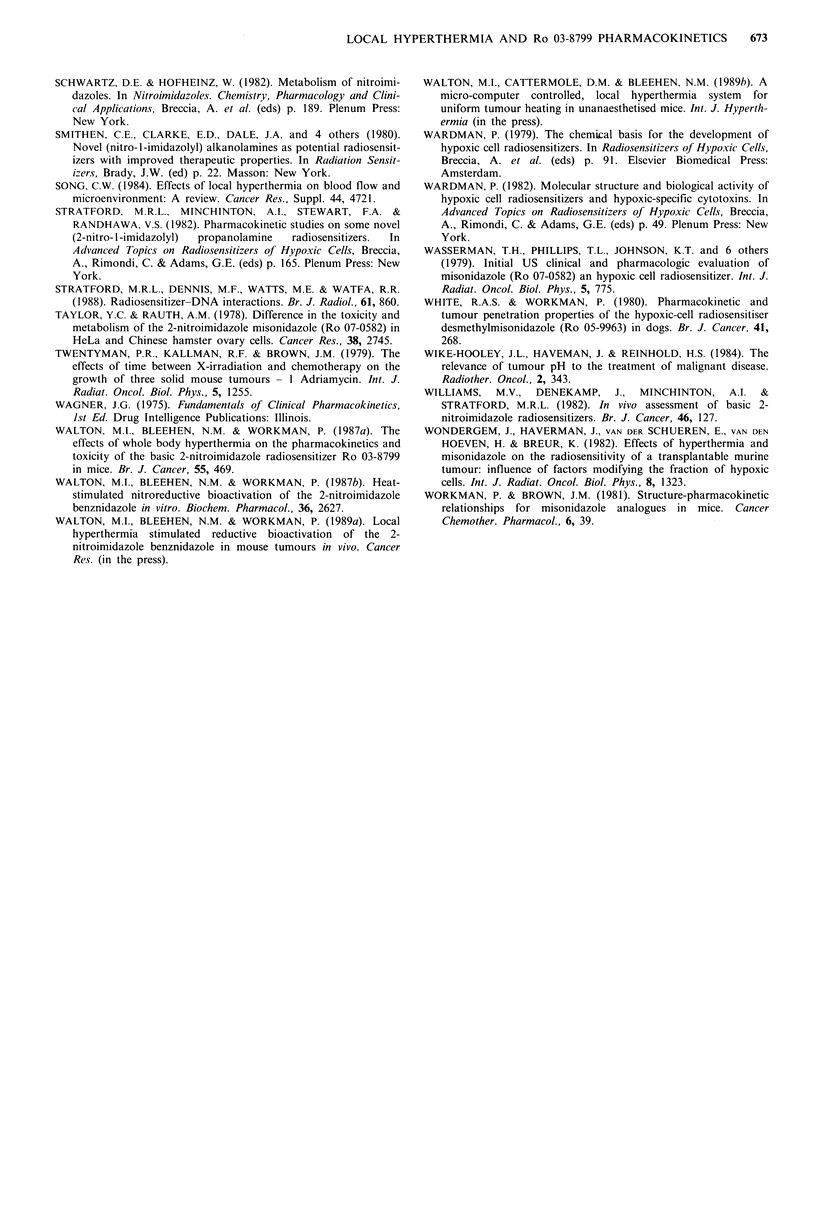# Effects of localised tumour hyperthermia on pimonidazole (Ro 03-8799) pharmacokinetics in mice.

**DOI:** 10.1038/bjc.1989.138

**Published:** 1989-05

**Authors:** M. I. Walton, N. M. Bleehen, P. Workman

**Affiliations:** MRC Clinical Oncology and Radiotherapeutics Unit, Cambridge, UK.

## Abstract

We have investigated the effects of localised tumour hyperthermia (LTH; 43.5 degrees C x 30 min) on the acute toxicity and pharmacokinetics of the hypoxic cell sensitizer pimonidazole (Ro 03-8799) in mice. There were three treatment groups: unrestrained controls, sham-treated and LTH treated mice. LTH had minimal effects on the acute toxicity (LD50/7d) of pimonidazole with no significant difference between the three treatment groups. Pharmacokinetic studies were carried out at the maximum tolerated dose (MTD; approximately 60% LD50) of 437 micrograms g-1 i.v. in plasma, brain and tumour. Sham tumour treatment consistently increased plasma drug concentrations compared to unrestrained controls but had minimal effects on the elimination t1/2. The AUC0-infinitive was increased by 35% and the plasma clearance decreased by 26%. By contrast, LTH had minimal effects on these parameters compared to sham treatment. Brain pimonidazole concentrations were increased in restrained mice (sham and LTH treatments) compared to unrestrained controls, but average brain/plasma ratios were similar in all three groups at between 400 and 500%. Sham tumour treatment markedly reduced peak tumour pimonidazole concentrations compared to unrestrained controls giving a 29% lower AUC0-180min. Average tumour/plasma ratios were reduced from 236 to 129%. The most important finding was that LTH further reduced pimonidazole tumour concentrations, giving a 31% lower AUC0-180 min compared to sham treated tumours. Tumour/plasma ratios for pimonidazole were reduced by 41%. Plasma exposure to the pimonidazole N-oxide metabolite, Ro 31-0313, was unaltered by LTH. The markedly reduced drug concentrations in heated tumours may be a result of hyperthermia-stimulated bioreductive drug activation.


*Br. J. Cancer* (1989), **59**, 667–673

© The Macmillan Press Ltd., 1989

### Effects of localised tumour hyperthermia on pimonidazole (Ro 03-8799) pharmacokinetics in mice

M.I. Walton, N.M. Bleehen & P. Workman

*MRC Clinical Oncology and Radiotherapeutics Unit, Hills Road, Cambridge CB2 2QH, UK.*

**Summary** We have investigated the effects of localised tumour hyperthermia (LTH; 43.5°C × 30 min) on the acute toxicity and pharmacokinetics of the hypoxic cell sensitizer pimonidazole (Ro 03-8799) in mice. There were three treatment groups: unrestrained controls, sham-treated and LTH treated mice. LTH had minimal effects on the acute toxicity ($LD_{50/7d}$) of pimonidazole with no significant difference between the three treatment groups. Pharmacokinetic studies were carried out at the maximum tolerated dose (MTD; ~60% $LD_{50}$) of 437 $\mu g\,g^{-1}$ i.v. in plasma, brain and tumour. Sham tumour treatment consistently increased plasma drug concentrations compared to unrestrained controls but had minimal effects on the elimination $t_{1/2}$. The $AUC_{0-\infty}$ was increased by 35% and the plasma clearance decreased by 26%. By contrast, LTH had minimal effects on these parameters compared to sham treatment. Brain pimonidazole concentrations were increased in restrained mice (sham and LTH treatments) compared to unrestrained controls, but average brain/plasma ratios were similar in all three groups at between 400 and 500%. Sham tumour treatment markedly reduced peak tumour pimonidazole concentrations compared to unrestrained controls giving a 29% lower $AUC_{0-180\,min}$. Average tumour/plasma ratios were reduced from 236 to 129%. The most important finding was that LTH further reduced pimonidazole tumour concentrations, giving a 31% lower $AUC_{0-180\,min}$ compared to sham treated tumours. Tumour/plasma ratios for pimonidazole were reduced by 41%. Plasma exposure to the pimonidazole N-oxide metabolite, Ro 31-0313, was unaltered by LTH. The markedly reduced drug concentrations in heated tumours may be a result of hyperthermia-stimulated bioreductive drug activation.

Hyperthermia (42–45°C) enhances the cytotoxic effects of radiation towards tumour cells both *in vitro* (Sapareto *et al.*, 1978) and *in vivo* (Mittal *et al.*, 1984). In addition local hyperthermia shows promise for clinical use (for reviews see Arcangeli *et al.*, 1988; Perez & Meyer, 1985).

Localised tumour hyperthermia (LTH) also potentiates the tumour cytotoxicity of certain drugs (Englehardt, 1987), including the electron-affinic 2-nitroimidazole radiosensitizer misonidazole (Bleehen *et al.*, 1977), in experimental tumours. The addition of misonidazole to LTH and X-rays can increase the local tumour control rates for this latter combination in mice (Overgaard, 1980; Wondergem *et al.*, 1982). Furthermore, a preliminary clinical study with the three modalities combined showed increased complete response rates in superficial neck node tumours compared to either radiotherapy alone or any pair of modalities together (Arcangeli *et al.*, 1980). Misonidazole is, however, unlikely to achieve optimal radiosensitization in man as a result of its dose-limiting neurotoxicity (e.g. Wasserman *et al.*, 1979; Dische *et al.*, 1982), and less neurotoxic and/or more potent analogues are undergoing development (Bartelink *et al.*, 1988).

Pimonidazole (α-[(2-nitro-1-imidazolyl)methyl]-1-piperidine-ethanol; Ro 03-8799), is a basic, lipophilic derivative exhibiting more efficient radiosensitization than misonidazole *in vitro* (Smithen *et al.*, 1980). It was hoped that its *p*Ka (8.7) would facilitate excretion in acidic urine and thereby reduce total-body exposure and hence toxicity (Wardman, 1979). The basicity of this molecule also appears to result in accumulation within cultured cells and their lysosomes and also binding to purified DNA (Stratford *et al.*, 1988; Dennis *et al.*, 1985). The drug also concentrates in tumours *in vivo*, resulting in tumour/plasma ratios in excess of 200% in mice and humans (Stratford *et al.*, 1982; Allen *et al.*, 1984; Roberts *et al.*, 1986). Similar ratios have been reported in several normal tissues as well (Williams *et al.*, 1982; Roberts *et al.*, 1986).

Tumour tissues commonly exhibit *p*Hs values below 7.4 (Wike–Hooley *et al.*, 1984). Factors contributing to acidosis include poor tumour blood perfusion resulting in reduced oxygen tension ($pO_2$), increased anaerobic glycolysis and decreased clearance of acidic metabolites (for a review see Calderwood & Dickson, 1983). LTH can reduce blood-flow in rodent tumours (Dudar & Jain, 1984; Song, 1984; Reinhold *et al.*, 1985) and has been shown to reduce both intracellular (Evanochko *et al.*, 1983) and extracellular (Wike–Hooley *et al.*, 1984) tumour pH, possibly through heat-stimulated anaerobic glycolysis and/or as a result of hyperthermia-induced disruptions in tumour blood-flow (Calderwood & Dickson, 1983; Reinhold *et al.*, 1985). A hyperthermia-induced drop in pH might be expected to increase tumour uptake of basic drugs such as pimonidazole from plasma (Wardman, 1982; Calderwood & Dickson, 1983), providing drug availability is not blood-flow limited. The selection of pimonidazole for use with hyperthermia may therefore be particularly appropriate. In addition, hyperthermia has also been shown to enhance the reductive bioactivation of nitro compounds *in vitro* (Walton *et al.*, 1987*b*; Olive, 1978).

In view of the enhanced cytotoxicity of nitroimidazole radiosensitisers with hyperthermia and the current clinical interest in radiotherapy combined with either radiosensitisers (e.g. Brown, 1986) or LTH (e.g. Overgaard, 1984), the possibility of combining all three modalities for the treatment of human cancer is an attractive one (Herman *et al.*, 1988). Because of the particular pharmacokinetic advantages which exist for pimonidazole compared to misonidazole and because of its continuing clinical development, we have investigated the effects of LTH on the acute toxicity, pharmacokinetics and metabolism of pimonidazole. In particular we have attempted to answer the following questions. (1) Does LTH alter the acute toxicity of pimonidazole? (2) Does LTH alter pimonidazole pharmacokinetics and selectively enhance tumour drug uptake? (3) Do the hyperthermia-drug interactions have a pharmacokinetic explanation and/or offer new information on the effects of LTH on drug metabolism? Such information should provide a useful guide to the potential efficacy of combining pimonidazole, LTH and radiotherapy in the clinic.

Correpondence: M.I. Walton.

Received 13 June 1988; and in revised form 4 November 1988.

### Materials and methods

*Mice and tumours*

Adult male C3H/He mice were obtained from our own breeding colony or from Olac Ltd (Bicester, UK). Mice were allowed food (PRD nuts; Labsure, Poole, Dorset) and water *ad libitum*, and were used at 25–33 g body weight.

The KHT sarcoma was grown in the gastrocnemius muscle of the right hind leg as previously described (Twentyman *et al.*, 1979). Mice were treated bearing tumours of 11–13 mm (orthodiagonal diameters) for pharmacokinetic experiments.

*Drug supply and administration*

Pimonidazole, its N-oxide Ro 31-0313 (α[(2-nitro-1-imidazolyl)methyl]-1-piperidine-ethanol 1-oxide) and the internal standard Ro 07-1902 (1-(2-nitroimidazol-1-yl)allyloxy propanol) were supplied by Roche (Welwyn Garden City, UK). Pimonidazole was supplied as the hydrochloride salt and all doses and concentrations are reported as the free base.

Drugs were administered in Hanks' balanced salt solution (HBSS, pH 7.4) at a fixed volume of 0.01 ml $g^{-1}$ body weight via the tail vein. Pimonidazole was injected over 35–40 s to avoid death by vascular shock (Williams *et al.*, 1982). Drugs were administered 5 min before LTH.

*Localised tumour hyperthermia (LTH)*

This was administered to unanaesthetised mice using a computer-controlled radiofrequency (RF)-waterbath heating system which produces uniform tumour heating with minimal increases in core (rectal) temperature (Walton *et al.*, 1989*b*). Approximately 2 min after drug administration mice were loaded into special perspex LTH jigs so that their shaved tumour-bearing legs were located between the RF electrodes. The LTH exposure was 43.5°C x 30 min, which is equal to a dose in equivalent minutes at 43°C (Eq 43) of 42.2 min (Dunlop *et al.*, 1984). This hyperthermia dose and treatment did not result in tumour oedema or normal tissue toxicity (see Walton *et al.*, 1989*b*). Groups of 3–4 mice were treated at the same time. Mice were allocated to three different protocols: (1) combined RF-waterbath LTH at 43.5°C x 30 min; (2) sham tumour treatment in perspex jigs without LTH but with the tumour bearing leg placed in a waterbath at 37°C; and (3) no treatment (unrestrained controls). After LTH and sham treatment, mice were rubbed dry with tissue paper and briefly warmed under a 40 W lamp to prevent post-treatment hypothermia.

*Acute toxicity*

The effect of LTH on the acute toxicity ($LD_{50/7d}$) of pimonidazole was determined using tumour-bearing animals as described elsewhere (Walton *et al.*, 1987*a*). Mice were allocated to one of the three different protocols described above. Graded doses of 524–900 $\mu g\, g^{-1}$ pimonidazole were given to groups of 4–5 mice with 3–4 doses per treatment. Mice were observed for 7 days post-treatment. $LD_{50/7d}$ values and confidence limits were derived using pooled data from three experiments by probit analysis, using the Generalised Interactive Modelling Program (GLIM) of the Royal Statistical Society of London.

*Sample analysis*

Samples were prepared and analysed as described in detail elsewhere (Walton *et al.*, 1987*a*). Briefly, whole blood was removed from anaesthetised animals and plasma obtained by centrifugation. Drugs were extracted into 10 volumes of acetonitrile containing the internal standard (Ro 07-1902) and taken to dryness before resuspension in 100 $\mu l$ of running buffer prior to HPLC analysis. After exsanguination animals were killed by cervical dislocation, and tumour and brain tissue was rapidly removed and snap-frozen on dry ice to prevent *ex vivo* metabolism. Tissue samples were homogenised (33% w/v) in distilled water before drug extraction and analysis as for plasma samples. All samples were handled on ice and stored at −20°C for up to 4 weeks before analysis.

Drug concentrations were assayed by reverse-phase, paired-ion, isocratic HPLC using a modified version of the method of Malcolm *et al.* (1983) as described elsewhere (Walton *et al.*, 1987*a*). Chromatography was carried out using columns and equipment supplied by Waters Ass. (Milford, MA, USA). Separations were performed on reverse-phase octadecylsilane (C18) Rad-Pak $\mu$ Bondapak columns (10 cm x 8 mm i.d., 10 $\mu m$ bead size) eluted isocratically with 17% acetonitrile in 0.2 M glycine/hydrochloric acid buffer containing 5 mM heptane sulphonic acid, pH 2.45, at a flow rate of 4.5 ml $min^{-1}$. Absorbance was monitored at 313 nm. Pimonidazole and Ro 31-0313 were identified by co-chromatography with authentic material and quantitated by peak-height ratio with reference to linear standard curves.

*Pharmacokinetic parameters*

Standard pharmacokinetic parameters (see Wagner, 1975) were calculated as described in detail elsewhere (White & Workman, 1980; Workman & Brown, 1981) using a one- or two-compartment model with curve stripping as appropriate. Lines of best fit were calculated by least squares linear regression analysis yielding half-lives with 95% confidence limits. Plasma area under the concentration × time curve ($AUC_{0-\infty}$) was calculated from the expression $AUC_{0-\infty} = C_0/k$, where $C_0$ is the concentration at time 0 and $k$ is the elimination rate constant, or from the equation $AUC_{0-\infty} = A/\alpha + B/\beta$, as appropriate. Tissue $AUC_{0-t}$ from time 0 to time $t$ was estimated by Simpson's rule.

*Statistics*

Significance levels were determined using the Student's $t$ test.

### Results

*Effects of LTH on pimonidazole acute toxicity*

Table I summarises the effects of LTH on the acute lethality ($LD_{50/7d}$) of pimonidazole in mice. LTH had no significant effect on pimonidazole toxicity compared to sham tumour treatment ($P > 0.5$), and drug plus sham tumour treatment was no more toxic than in unrestrained controls ($P > 0.05$). Deaths were either rapid and convulsive, occurring during or immediately after drug injection and before jig loading, or non-convulsive and occurring during the subsequent 24 h. No deaths resulted during the period of either LTH or sham tumour treatments.

**Table I** Effects of LTH on the acute *Toxicity $LD_{50/7d}$($\mu g\, g^{-1}$)* C3H mice.

| *Toxicity $LD_{50/7d}$($\mu g\, g^{-1}$)* | | |
|---|---|---|
| *Unrestrained control* | *Sham treatment* | *LTH treatment* |
| 758 (720–798) | 775 (726–829) | 710 (551–829) |

Results were obtained using pooled data from three independent experiments with 4–5 mice per dose and 3–4 doses per experiment. 95% confidence limits are shown in parentheses.

Subsequent pharmacokinetic studies were carried out at the previously determined maximum tolerated dose (~60% $LD_{50/7d}$) in unrestrained control mice of 437 $\mu g\, g^{-1}$ (see Walton *et al.*, 1987*a*) as this was similar to the MTD for unrestrained mice determined here ($P > 0.2$). Mice were given either pimonidazole alone or pimonidazole combined with LTH or sham tumour treatment. No deaths occurred at this dose in any treatment group (total number of mice = 46).

*Effects of LTH and pimonidazole on tumour and core temperatures*

High doses of 2-nitroimidazoles cause hypothermia in mice (Gomer & Johnson, 1979), and the dose of pimonidazole used in these LTH experiments has previously been shown to decrease mouse rectal temperature by 2–3°C for up to 1 h after administration (Walton *et al.*, 1987*a*). In view of this, we determined the effects of LTH applied 5 min after 437 $\mu g\, g^{-1}$ pimonidazole i.v. on tumour and rectal temperatures.

Figure 1 shows that central tumour temperatures reached 43.5°C about 4 min after the start of heating and were maintained to within 0.2–0.3°C (range) of the target temperature for the majority of the treatment. This hyperthermia treatment (43.5°C × 35 min) gave a total thermal dose measured in equivalent minutes at 43°C (Eq 43) of 40.8 ± 0.35 min (mean ± 2 s.e.; $n = 4$), which represents 97% of the prescribed dose. Average core temperatures increased slightly over the heating period from about 36 to 37°C, and were consistently 1°C higher than in unrestrained control mice given this dose of pimonidazole alone (Walton *et al.*, 1987*a*).

*Effects of LTH on pimonidazole pharmacokinetics*

*Plasma pharmacokinetics.* Pimonidazole plasma clearance was biphasic in unrestrained control mice (Figure 2a). The initial α-phase was short ($t_{1/2}\alpha \sim 2.5$ min) and essentially complete within the first 10 min after drug administration, while the terminal β-phase was much longer with a $t_{1/2}\beta$ (with 95% confidence limits) of 31.3 (28.9–34) min. As the error in treating these data as monoexponential was small (5.7%) they were fitted to a one-compartment model in accordance with the guidelines of Dvorchik & Vesell (1978).

Figure 2a shows that plasma drug concentrations were consistently higher in mice with sham-treated tumours compared to unrestrained control mice, with significant increases at 90, 120 and 180 min ($P < 0.05$ in each case). The elimination $t_{1/2}$ were highly comparable ($P > 0.5$) but sham tumour treatment reduced the apparent Vd by 18% compared to the value in unrestrained controls ($P < 0.05$).

**Figure 1** The effects of LTH (43.5°C × 30 min) started 5 min after 437 $\mu g\, g^{-1}$ pimonidazole i.v. on the central tumour (○) and rectal (□) temperatures in C3H mice bearing KHT leg tumours. Results are mean ± 2 s.e. for a typical LTH treatment using four mice. Similar results were obtained in repeat experiments.

**Figure 2** The effects of LTH (43.5°C × 30 min) on the pharmacokinetics of pimonidazole in (**a**) plasma, (**b**) brain and (**c**) tumour tissue of C3H mice administered 437 $\mu g\, g^{-1}$ pimonidazole i.v. Symbols: △ unrestrained control mice; ○ sham tumour treated mice; ● LTH treated mice. Results are mean ± 2 s.e. Pooled data from two independent experiments each involving 5–8 time points with 3–4 mice per point. Lines fitted by least-squares linear regression analysis.

The $AUC_{0-\infty}$ was increased by 35% and the plasma clearance ($P_{Cl}$) correspondingly reduced by 26% in sham tumour treated compared to unrestrained control mice.

By comparison, LTH did not further affect plasma pimonidazole concentrations compared to sham tumour treatment (Figure 2a), and the respective elimination $t_{1/2}$s were also very similar (Table II; $P>0.5$). In addition Vd, $AUC_{0-\infty}$ and $P_{Cl}$ were minimally altered by LTH compared to sham tumour treatment (Table II).

*Brain pharmacokinetics.* Brain pimonidazole concentrations reached a peak of around $340\,\mu g\,g^{-1}$ in both unrestrained controls and mice with sham-treated tumours, though it was much broader in the latter group (Figure 2b). Brain pimonidazole concentrations were consistently higher during the elimination phase in mice bearing sham-treated tumours compared to unrestrained controls, in accord with their higher plasma drug concentrations; this difference was significant at 90, 120 and 180 min ($P<0.05$, $P<0.01$ and $P<0.05$ respectively). There was no difference between brain elimination $t_{1/2}$ for unrestrained control and sham tumour treated mice ($P>0.3$), but $AUC_{0-180\,min}$ values were reproducibly increased by an average 13% in the latter group (Table II).

LTH had minimal effects on brain pimonidazole pharmacokinetics compared to sham tumour treatment. LTH did not significantly alter either brain drug concentrations or the elimination $t_{1/2}$ ($P>0.2$). $AUC_{0-180\,min}$ values were very similar.

After equilibration at 60 min, brain/plasma ratios were similar in all three treatment groups at between 360 and 628% (range). For example brain/plasma ratios at 50 or 60 min were $402\pm113$, $393\pm66.6$ and $364\pm60.5$% (mean $\pm 2$ s.e.; $n=6$) in unrestrained control, sham tumour and LTH treated mice respectively, values in good agreement with previous work (Walton *et al.*, 1987*a*). Average steady-state brain/plasma ratios (mean $\pm 2$ s.e.; $n=22$) were very similar at $498\pm64$, $454\pm34$ and $433\pm38$% for unrestrained control mice and those bearing sham- and LTH-treated tumours respectively ($P>0.05$).

*Tumour pharmacokinetics.* Tumour pimonidazole concentrations in unrestrained control mice reached a peak of about $250\,\mu g\,g^{-1}$ 20–30 min after administration (Figure 2c). The elimination $t_{1/2}$ was 31.6 min and the $AUC_{0-180\,min}$ was $441\,\mu g\,g^{-1}\,h$ (Table II). Sham-treated tumours had a 2-fold lower peak concentration, the difference being highly significant at 20 min ($P<0.01$). However, the levels became similar during the terminal elimination phase (Figure 2c). Sham tumour treatment had minimal effects on tumour $t_{1/2}$ ($P>0.1$), but reduced the $AUC_{0-180\,min}$ by 29% compared to the value in unrestrained control tumours.

LTH had a very marked effect on tumour pimonidazole concentrations compared to sham-treated tumours (Figure 2c). Locally heated tumours had a lower peak drug concentration and exhibited consistently reduced drug levels over the whole time course compared to sham-treated, giving significantly different zero time intercepts for each treatment ($P<0.05$). This reduction in pimonidazole concentrations in heated tumours occurred despite the higher plasma drug concentrations in these mice. The average $AUC_{0-180\,min}$ was decreased by 31% compared to sham-treated tumours. The tumour $AUC_{0-180\,min}$ was decreased by 35 and 26% in the two independent experiments. LTH did not significantly alter pimonidazole tumour elimination $t_{1/2}$ which were similar in all three treatment groups ($P>0.1$).

Table III shows the tumour/plasma ratios from the above data. Tumour/plasma ratios in unrestrained control mice consistently exceeded 200% after equilibration at 20 min, in good agreement with other values (Williams *et al.*, 1982; Walton *et al.*, 1987*a*). Ratios in sham-treated tumours were consistently less than those in unrestrained controls. Average ratios were stable from 10–180 min and exceeded 100% after 35 min but were all <175%. The overall mean ratios were significantly reduced in sham-treated tumours from $236\pm16.9$ to $129\pm17$% (mean $\pm 2$ s.e.; $n=31$ and 40 respectively; $P<0.001$).

LTH had even more pronounced effects on tumour drug concentrations compared to sham tumour treatment. LTH markedly reduced tumour/plasma ratios over the whole time course with significant decreases at 35, 120 and 180 min (Table III). In addition, steady-state ratios never exceeded 100% in heated tumours and the average tumour/plasma ratio over this period was significantly reduced from $129\pm17$ to $87.8\pm10.9$% (mean $\pm 2$ s.e.; $n=40$; $P<0.001$).

**Table II** Summary of the effects of LTH on the pharmacokinetics of pimonidazole ($437\,\mu g\,g^{-1}$ i.v.) in plasma, brain and KHT tumour of C3H mice

| | *Treatment* | | |
|---|---|---|---|
| | *Unrestrained control* | *Sham treatment* | *LTH treatment* |
| *Plasma* | | | |
| $t_{1/2}$ (min) | 31.3 (28.9–34) | 34.7 (32.3–37.5) | 36.0 (32.9–39.8) |
| $Vd_{ext}$ ($ml\,g^{-1}$) | 2.59[a] (2.21–3.05) | 2.12 (1.86–2.41) | 2.30 (1.97–2.69) |
| $AUC_{0-\infty}$ ($\mu g\,ml^{-1}\,h$) | 127 (135, 127) | 172 (168, 175) | 165 (145, 184) |
| $P_{Cl}$ ($ml\,g^{-1}\,h^{-1}$) | 3.44 (3.23, 3.44) | 2.54 (2.60, 2.50) | 2.65 (3.01, 2.38) |
| *Brain* | | | |
| $t_{1/2}$ (min) | 31.6 (29–34.6) | 33.9 (30.7–37.8) | 38.4 (32.9–46.1) |
| $AUC_{0-180\,min}$ ($\mu g\,g^{-1}\,h$) | 441 (434, 477) | 529 (503, 586) | 499 (393, 617) |
| *Tumour* | | | |
| $t_{1/2}$ (min) | 31.6 (29.1–33.3) | 35.2 (30.7–41.2) | 33.6 (30.1–37.9) |
| $AUC_{0-180\,min}$ ($\mu g\,g^{-1}\,h$) | 268 (263, 299) | 191 (235, 157) | 132 (152, 116) |

Parameters were derived using pooled data from two independent experiments. These were fitted to a one-compartment model. 95% confidence limits are shown in parentheses for $t_{1/2}$ and Vd values. Figures in parentheses for AUC and $P_{Cl}$ represent the values determined in two independent experiments (A and B, respectively). Each experiment involved 7–8 time points with 3–9 mice per point. [a]$P<0.05$ significantly different from sham treatment. The unrestrained control $t_{1/2}$ and the LTH $t_{1/2}$ and Vd values were not significantly different from sham-treatment values ($P>0.05$).

### *Effects of LTH on pimonidazole N-oxidation*

Figure 3 shows the plasma concentrations of Ro 31-0313, the N-oxide metabolite of pimonidazole, after pimonidazole administration alone, or combined with LTH or sham tumour treatment. Peak plasma concentrations of Ro 31-0313 were increased during LTH compared to sham-treated mice, e.g. by 34% from 9.77 to $13.1\,\mu g\,ml^{-1}$ at 20 min ($P<0.05$). However, the plasma $AUC_{0-180\,min}$ was essentially unaffected. The values in the two independent experiments were 19.3 and 23.5 compared to 18.4 and $22.5\,\mu g\,ml^{-1}\,h$ for LTH and sham-treated mice respectively. Plasma Ro 31-0313 concentrations in unrestrained control mice were consistently lower than for sham tumour treated mice. This resulted in a 25% lower $AUC_{0-180\,min}$ of $14.9\,\mu g\,ml^{-1}\,h$, the values in two independent experiments being 14.6 and $16.1\,\mu g\,ml^{-1}\,h$.

Tumour and brain Ro 31-0313 levels were frequently at or just above the lower limit of detection ($\sim 1\,\mu g\,g^{-1}$). Maximum tumour Ro 31-0313 concentrations did not exceed $7\,\mu g\,g^{-1}$ giving a tumour/plasma ratio of 43%, while maximum brain concentrations did not exceed $3.5\,\mu g\,g^{-1}$ corresponding to a brain/plasma ratio of 63%.

**Table III** Effects of LTH on the tumour/plasma ratios for pimonidazole (437 $\mu g\,g^{-1}$ i.v.) in C3H mice

| *Time after drug administration (min)* | | *Tumour/plasma (%)* | | |
|---|---|---|---|---|
| | | *Unrestrained control* | *Sham treatment* | *LTH treatment* |
| LTH | 10 | $179 \pm 20.8^b$ ($n=4$) | $98.3 \pm 26.3$ ($n=6$) | $91.2 \pm 52.8$ ($n=6$) |
| LTH | 20 | $219 \pm 11.3^b$ ($n=9$) | $96.6 \pm 11.4$ ($n=6$) | $87.9 \pm 18.9$ ($n=6$) |
| LTH | 35 | ND | $155 \pm 42.7$ ($n=6$) | $93.1 \pm 18.9^a$ ($n=6$) |
| | 50 | $266 \pm 35.3$ ($n=6$) | ND | ND |
| | 60 | ND | $118 \pm 58.1$ ($n=6$) | $87.6 \pm 39.8$ ($n=6$) |
| | 90 | $250 \pm 70.9^a$ ($n=6$) | $136 \pm 55.4$ ($n=6$) | $88.7 \pm 21.0$ ($n=6$) |
| | 120 | $238 \pm 20.0^a$ ($n=6$) | $175 \pm 42.4$ ($n=6$) | $82.9 \pm 20.2^b$ ($n=6$) |
| | 180 | $205 \pm 19.5^b$ ($n=4$) | $124 \pm 25.8$ ($n=4$) | $81.1 \pm 20.1^a$ ($n=4$) |

Results are mean $\pm 2$ s.e. of $n$ values which were derived using pooled data from two independent experiments. $^aP<0.05$ and $^bP<0.01$ significantly different from sham treatment. ND, Not determined.

**Figure 3** Plasma concentrations of the N-oxide metabolite Ro 31-0313, after pimonidazole administration (437 $\mu g\,g^{-1}$ i.v.) in C3H mice. Symbols: ▽ unrestrained control mice; ◇ sham tumour treated mice; and ◆ LTH treated mice. Results are mean $\pm 2$ s.e. using pooled data from two independent experiments with 4–9 mice per point.

### Discussion

The toxicological results show that LTH (43.5°C × 30 min) does not increase the acute toxicity of pimonidazole in mice whose core temperatures were maintained at 36–38°C. This is in marked contrast to the situation with whole-body hyperthermia (WBH; 41°C × 35 min) where pimonidazole acute toxicity was increased 3-fold (Walton *et al.*, 1987*a*). Misonidazole and etanidazole (SR 2508) lethality were also enhanced by WBH (Bleehen *et al.*, 1988). Other investigators have observed increased misonidazole acute lethality in mice treated with LTH (Overgaard, 1979; Bleehen *et al.*, 1977) but rectal temperatures approached 41°C towards the end of heating. Cisplatinum acute lethality has also been shown to increase with LTH (44°C × 60 min) in rats (Mella, 1985) and systemic cooling reduced this toxicity. If hyperthermia-enhanced acute drug toxicity in mice results from elevated systemic temperatures, as appears to be the case, then careful body temperature regulation will be required in order to minimise these effects during LTH.

The pharmacokinetic results show that sham tumour treatment resulted in elevated plasma drug concentrations compared to unrestrained controls without affecting the elimination $t_{1/2}$. Consequently, mice bearing sham-treated tumours had a lower Vd, higher $AUC_{0-\infty}$ and a correspondingly slower $P_{Cl}$ than unrestrained controls. Furthermore, the results also show that LTH has no significant effect on pimonidazole plasma concentrations or $t_{1/2}$ in mice. It has minimal effects on plasma $AUC_{0-\infty}$ or $P_{Cl}$, though Vd was slightly increased. This difference between restrained mice (both LTH and sham tumour treated) compared to unrestrained control mice may result from stress-impaired renal drug clearance under the former conditions.

Unrestrained control mice had slightly lower brain pimonidazole levels than sham tumour treated animals. This was almost certainly due to their lower plasma pimonidazole concentrations, as equilibration with plasma was comparable resulting in very similar brain/plasma ratios. Pimonidazole concentrated in brain tissue to a similar degree in sham tumour and LTH treated mice with brain/plasma ratios of between 400 and 600% (range).

Tumour/plasma ratios in unrestrained control mice equilibrated at between 205 and 266%, values comparable though slightly lower than in previous reports (Williams *et al.*, 1982; Walton *et al.*, 1987*a*). Sham-treated tumour pimonidazole drug concentrations were slightly less than those in unrestrained control tumours, giving nearly a 2-fold lower average tumour/plasma ratio of 129%. This reduction in pimonidazole concentrations in sham-treated tumours could have arisen through altered blood perfusion or metabolism in the tumour-bearing leg as a result of stress or direct restriction. If so, then not all handling artifacts have been removed from this system, and LTH might be more efficacious if applied after peak tumour drug levels have been attained.

Perhaps the most consistent and important effect of LTH was its ability substantially to reduce tumour concentrations of pimonidazole over the whole time course, giving a 31% lower $AUC_{0-180\,min}$ in heated tumours compared to sham-treated ones. Tumour/plasma ratios were also significantly lower in locally heated tumours with a 41% decrease in the average steady-state ratio. This finding is similar to that reported for MISO and LTH (44°C × 60 min; Honess *et al.*, 1980) where nitroimidazole concentrations in heated tumours were reduced by up to 70% giving a 25% lower $AUC_{0-8h}$ and a reduction of up to 65% in heated tumour/plasma ratios compared to controls. Decreased tumour/plasma ratios in heated tumours may be the result of several factors. There is evidence that LTH can decrease or abolish tumour blood-flow (Bicher *et al.*, 1980; Dudar & Jain, 1984; Song, 1984). This might lead to reduced drug supply to the tumour and consequently lower tumour drug uptake. Moreover, if hyperthermia-decreased tumour pH occurs as a result of the accumulation of metabolic waste products through heat-impaired tumour blood perfusion, then LTH might not increase tumour uptake of basic drugs from the plasma because of limited drug supply.

An alternative explanation is that LTH stimulates the nitroreductive bioactivation of pimonidazole to non-UV absorbing metabolites (Schwartz & Hofheinz, 1982), a process strongly implicated in the hypoxic cytotoxicity of 2-nitroimidazoles (Taylor & Rauth, 1978; Rauth, 1984). Further support for this hypothesis comes from our studies where LTH increased KHT tumour concentrations of the reduced amine metabolite of the 2-nitroimidazole benznidazole (Walton *et al.*, 1989*a*) and other work where LTH (43°C × 60 min) increased concentrations of reduced adriamycin metabolites, especially aglycones, in murine tumours (Magin *et al.*, 1980). Adriamycin aglycone production in rat liver slices is also enhanced by elevated temperatures under anaerobic conditions *in vitro* (Dodion *et al.*, 1986). As pimonidazole is stable for at least 2 h at 43.5°C in sodium phosphate buffer (pH 7.4) and mouse plasma (Walton *et al.*, unpublished data) heat-enhanced spontaneous chemical degradation can be ruled out.

LTH increased peak plasma concentrations of the pimonidazole N-oxide metabolite Ro 31-0313 during heating. This may reflect an increase in oxidative metabolism and/or decreased plasma clearance of the metabolite. This finding is in marked contrast to WBH where pimonidazole N-oxidation was greatly elevated in mouse plasma during and after the heating period (Walton *et al.*, 1987*a*). Previous studies with MISO have shown that LTH decreases peak plasma concentrations of the O-demethylated metabolite of misonidazole, though the metabolite AUCs were comparable in heated and control mice (Honess *et al.*, 1980).

In summary, LTH did not alter the acute lethality of pimonidazole in mice. It had minimal effects on plasma and brain pimonidazole pharmacokinetics, and only slightly increased plasma N-oxide concentrations. However, tumour drug concentrations were significantly decreased by LTH, possibly through heat-stimulated reductive bioactivation. These results show that LTH has a complex effect on pimonidazole tumour pharmacokinetics and metabolism. Further studies are required to elucidate the effects of hyperthermia on oxidative and reductive metabolism, particularly in view of the cytotoxic potential of the latter reaction in hypoxic cells and its possible enhancement in locally heated tumours. Combinations of LTH with hypoxic-cell targeted radiosensitisers and other bioreductive drugs are likely to require further addition of radiation or appropriate drugs to eliminate the oxic cell population.

We thank Dr Carey Smithen (Roche, Welwyn Garden City, UK) for supplies of the nitroimidazoles. We also than Dr Max Parmar for help with the statistical analyses and we are also grateful to Mrs J. Shaw for care of the animals. M. Walton thanks the Cancer Research Campaign for a research studentship award.

### References

ALLEN, J.G., DISCHE, S., LENOX-SMITH, I., MALCOLM, S.L. & SAUNDERS, M.I. (1984). The pharmacokinetics of a new radiosensitizer, Ro 03-8799 in humans. *Eur. J. Clin. Pharmacol.*, **27**, 483.

ARCANGELI, G., CIVIDALLI, A., LOVISOLO, G. and 4 others (1980). Effectiveness of local hyperthermia in association with radiotherapy or chemotherapy: comparison of multimodality treatments on multiple neck node metastases. In *Hyperthermia in Radiation Oncology*, Arcangeli G. & Mauro F. (eds) p. 257. Masson: Milan.

ARCANGELI, G., OVERGAARD, J., GONZALEZ, D.G. & SHRIVASTAVA, P.N. (1988). Hyperthermia trials. *Int. J. Radiat. Oncol. Biol. Phys.*, **14**, S93.

BARTELINK, H., BLEEHEN, N.M. & PHILLIPS, T.L. (1988). Chemical modifiers. *Int. J. Radiat. Oncol. Biol. Phys.*, **14**, S39.

BICHER, H.I., HETZEL, F.W., SANDHU, T.S. and 4 others (1980). Effects of hyperthermia on normal and tumour microenvironment. *Radiology*, **137**, 523.

BLEEHEN, N.M., HONESS, D.J. & MORGAN, J.E. (1977). Interaction of hyperthermia and the hypoxic cell sensitiser Ro 07-0582 on the EMT6 mouse tumour. *Br. J. Cancer*, **35**, 229.

BLEEHEN, N.M., WALTON, M.I. & WORKMAN, P. (1988). The interaction of hyperthermia with hypoxic cell sensitizers. *Recent Results Cancer Res.*, **109**, 136.

BROWN, J.M. (1986). Chemical modifiers of cancer treatment. *Int. J. Radiat. Oncol. Biol. Phys.*, **12**, 1.

CALDERWOOD, S.K. & DICKSON, J.A. (1983). pH and tumour response to hyperthermia. *Adv. Radiat. Biol.*, **10**, 135.

DENNIS, M.F., STRATFORD, M.R.L., WARDMAN, P. & WATTS, M.E. (1985). Cellular uptake of misonidazole and analogues with acidic or basic functions. *Int. J. Radiat. Biol.*, **47**, 629.

DISCHE, S., SAUNDERS, M.I., ANDERSON, P., STRATFORD, M.R.L. & MINCHINTON, A.M. (1982). Clinical experience with nitroimidazoles as radiosensitizers. *Int. J. Radiat. Oncol. Biol. Phys.*, **8**, 335.

DODION, P., RIGGS, C.E., ALKMAN, S.R. & BACHUR, N.R. (1986). Effects of hyperthermia on the *in vitro* metabolism of doxorubicin. *Cancer Treat. Rep.*, **70**, 625.

DUDAR, T.E. & JAIN, R.K. (1984). Differential response of normal and tumour microcirculation to hyperthermia. *Cancer Res.*, **44**, 605.

DUNLOP, P.R.C., DICKINSON, R.S., HAND, J.W. & FIELD, S.B. (1984). The use of thermal dose in the clinical application of localised hyperthermia. In *Hyperthermic Oncology*, Overgaard, J. (ed) p. 187. Taylor and Francis: London.

DVORCHIK, B.H. & VESELL, E.S. (1978). Significance of error associated with use of the one-compartment formula to calculate clearance of thirty-eight drugs. *Clin. Pharmacol. Ther.*, **23**, 617.

ENGLEHARDT, R. (1987). Hyperthermia and drugs. *Recent Results Cancer Res.*, **104**, 133.

EVANOCHKO, W.T., NG, T.C., LILLY, M.B. and 4 others (1983). *In vivo* $^{31}$P NMR study of the metabolism of murine mammary 16/C adenocarcinoma and its response to chemotherapy, X-radiation and hyperthermia. *Proc. Natl Acad. Sci, USA*, **80**, 334.

GOMER, C.J. & JOHNSON, R.J. (1979). Relationship between misonidazole toxicity and core temperature in C3H mice. *Radiat. Res.*, **78**, 329.

HERMAN, T.S., TEICHER, B.A., JOCHELSON, M., CLARK, J., SVENSSON, G. & COLEMAN, C.N. (1988). Rationale for use of local hyperthermia with radiation therapy and selected anticancer drugs in locally advanced human malignancies. *Int. J. Hyperthermia*, **4**, 143.

HONESS, D.J., WORKMAN, P., MORGAN, J.E. & BLEEHEN, N.M. (1980). Effects of local hyperthermia on the pharmacokinetics of misonidazole in the anaesthetised mouse. *Br. J. Cancer*, **41**, 529.

MAGIN, R.L., LYSYK, R.L. & LITTERST, C.L. (1980). Distribution of adriamycin in mice under conditions of local hyperthermia which improve systemic drug therapy. *Cancer Treat. Rep.*, **64**, 203.

MALCOLM, S.L., LEE, A. & GROVES, J.K. (1983). High-performance liquid chromatographic analysis of the new hypoxic cell radiosensitizer, Ro 03-8799, in biological samples. *J. Chromatogr.*, **273**, 327.

MELLA, O. (1985). Combined hyperthermia and *cis*-diamminedichloroplatinum in BD IX rats with transplanted $BT_4A$ tumours. *Int. J. Hyperthermia*, **1**, 171.

MITTAL, B., EMANI, B., SAPARETO, S.A., TAYLOR, F.H. & ABRATH, F.G. (1984). Effects of sequencing of the total course of combined hyperthermia and radiation on the RIF-1 murine tumour. *Cancer*, **54**, 2889.

OLIVE, P. (1978). Enhanced metabolic reduction and cytotoxicity of nitrofurazone at high temperatures. *Cancer Biochem. Biophys.*, **2**, 155.

OVERGAARD, J. (1979). Effect of local hyperthermia on the acute toxicity of misonidazole in mice. *Br. J. Cancer*, **39**, 96.

OVERGAARD, J. (1980). Effects of misonidazole and hyperthermia on the radiosensitivity of a C3H mouse mammary carcinoma and its surrounding normal tissue. *Br. J. Cancer*, **41**, 10.

OVERGAARD, J. (1984). *Hyperthermic Oncology*. Taylor and Francis: London.

PEREZ, C.A. & MEYER, J.L. (1985). Clinical experience with localised hyperthermia and irradiation. In *Hyperthermic Oncology*, Overgaard, J. (ed) p. 181. Taylor and Francis: London.

RAUTH, A.M. (1984). Pharmacology and toxicology of sensitizers: mechanism studies. *Int. J. Radiat. Oncol. Biol. Phys.*, **10**, 1293.

REINHOLD, H.S., WIKE-HOOLEY, J.L., VAN DEN BERG-BLOK (1985). Environmental factors, blood flow and microcirculation. In *Hyperthermic Oncology*, Overgaard, J. (ed) p. 41. Taylor and Francis: London.

ROBERTS, J.T., BLEEHEN, N.M., WALTON, M.I. & WORKMAN, P. (1986). A clinical Phase 1 toxicity study of Ro 03-8799: plasma, urine, tumour and normal brain pharmacokinetics. *Br. J. Radiol.*, **59**, 107.

SAPARETO, S.A., HOPWOOD, L.E. & DEWEY, W.C. (1978). Combined effects of X-irradiation and hyperthermia on CHO cells for various temperatures and orders of application. *Radiat. Res.*, **73**, 221.

SCHWARTZ, D.E. & HOFHEINZ, W. (1982). Metabolism of nitroimidazoles. In *Nitroimidazoles. Chemistry, Pharmacology and Clinical Applications*, Breccia, A. *et al.* (eds) p. 189. Plenum Press: New York.

SMITHEN, C.E., CLARKE, E.D., DALE, J.A. and 4 others (1980). Novel (nitro-1-imidazolyl) alkanolamines as potential radiosensitizers with improved therapeutic properties. In *Radiation Sensitizers*, Brady, J.W. (ed) p. 22. Masson: New York.

SONG, C.W. (1984). Effects of local hyperthermia on blood flow and microenvironment: A review. *Cancer Res.*, Suppl. 44, 4721.

STRATFORD, M.R.L., MINCHINTON, A.I., STEWART, F.A. & RANDHAWA, V.S. (1982). Pharmacokinetic studies on some novel (2-nitro-1-imidazolyl) propanolamine radiosensitizers. In *Advanced Topics on Radiosensitizers of Hypoxic Cells*, Breccia, A., Rimondi, C. & Adams, G.E. (eds) p. 165. Plenum Press: New York.

STRATFORD, M.R.L., DENNIS, M.F., WATTS, M.E. & WATFA, R.R. (1988). Radiosensitizer–DNA interactions. *Br. J. Radiol.*, **61,** 860.

TAYLOR, Y.C. & RAUTH, A.M. (1978). Difference in the toxicity and metabolism of the 2-nitroimidazole misonidazole (Ro 07-0582) in HeLa and Chinese hamster ovary cells. *Cancer Res.*, **38,** 2745.

TWENTYMAN, P.R., KALLMAN, R.F. & BROWN, J.M. (1979). The effects of time between X-irradiation and chemotherapy on the growth of three solid mouse tumours – 1 Adriamycin. *Int. J. Radiat. Oncol. Biol. Phys.*, **5,** 1255.

WAGNER, J.G. (1975). *Fundamentals of Clinical Pharmacokinetics, 1st Ed.* Drug Intelligence Publications: Illinois.

WALTON, M.I., BLEEHEN, N.M. & WORKMAN, P. (1987*a*). The effects of whole body hyperthermia on the pharmacokinetics and toxicity of the basic 2-nitroimidazole radiosensitizer Ro 03-8799 in mice. *Br. J. Cancer*, **55,** 469.

WALTON, M.I., BLEEHEN, N.M. & WORKMAN, P. (1987*b*). Heat-stimulated nitroreductive bioactivation of the 2-nitroimidazole benznidazole *in vitro*. *Biochem. Pharmacol.*, **36,** 2627.

WALTON, M.I., BLEEHEN, N.M. & WORKMAN, P. (1989*a*). Local hyperthermia stimulated reductive bioactivation of the 2-nitroimidazole benznidazole in mouse tumours *in vivo*. *Cancer Res.* (in the press).

WALTON, M.I., CATTERMOLE, D.M. & BLEEHEN, N.M. (1989*b*). A micro-computer controlled, local hyperthermia system for uniform tumour heating in unanaesthetised mice. *Int. J. Hyperthermia* (in the press).

WARDMAN, P. (1979). The chemical basis for the development of hypoxic cell radiosensitizers. In *Radiosensitizers of Hypoxic Cells*, Breccia, A. *et al.* (eds) p. 91. Elsevier Biomedical Press: Amsterdam.

WARDMAN, P. (1982). Molecular structure and biological activity of hypoxic cell radiosensitizers and hypoxic-specific cytotoxins. In *Advanced Topics on Radiosensitizers of Hypoxic Cells*, Breccia, A., Rimondi, C. & Adams, G.E. (eds) p. 49. Plenum Press: New York.

WASSERMAN, T.H., PHILLIPS, T.L., JOHNSON, K.T. and 6 others (1979). Initial US clinical and pharmacologic evaluation of misonidazole (Ro 07-0582) an hypoxic cell radiosensitizer. *Int. J. Radiat. Oncol. Biol. Phys.*, **5,** 775.

WHITE, R.A.S. & WORKMAN, P. (1980). Pharmacokinetic and tumour penetration properties of the hypoxic-cell radiosensitiser desmethylmisonidazole (Ro 05-9963) in dogs. *Br. J. Cancer*, **41,** 268.

WIKE-HOOLEY, J.L., HAVEMAN, J. & REINHOLD, H.S. (1984). The relevance of tumour pH to the treatment of malignant disease. *Radiother. Oncol.*, **2,** 343.

WILLIAMS, M.V., DENEKAMP, J., MINCHINTON, A.I. & STRATFORD, M.R.L. (1982). *In vivo* assessment of basic 2-nitroimidazole radiosensitizers. *Br. J. Cancer*, **46,** 127.

WONDERGEM, J., HAVERMAN, J., VAN DER SCHUEREN, E., VAN DEN HOEVEN, H. & BREUR, K. (1982). Effects of hyperthermia and misonidazole on the radiosensitivity of a transplantable murine tumour: influence of factors modifying the fraction of hypoxic cells. *Int. J. Radiat. Oncol. Biol. Phys.*, **8,** 1323.

WORKMAN, P. & BROWN, J.M. (1981). Structure-pharmacokinetic relationships for misonidazole analogues in mice. *Cancer Chemother. Pharmacol.*, **6,** 39.